# Automation of a spectrophotometric method for measuring L -carnitine in human blood serum

**DOI:** 10.1155/S1463924698000042

**Published:** 1998

**Authors:** Amparo Galan, Anna Padros, Marta Arambarri, Silvia Martin

**Affiliations:** Clinical Biochemistry Service Hospital Universitario ‘Germans Trias i Pujol,’ Badalona, Barcelona Spain

## Abstract

A spectrometric method for the determination of L-carnitine has been developed based on the reaction of the 5,$5^{\prime }$ dithiobis-(2-nitrobenzoic) acid (DTNB) and adapted to a Technicon RA-2000 automatic analyser Química Farmacéutica Bayer, S.A.). The detection limit of the method is 13.2 μmol/l, with a measurement interval ranging from 30 to 320 μmoll1. Imprecision and accuracy are good even at levels close to the detection limit (coeffcient of variation of 5.4% for within-run imprecision for a concentration of 35 μmol/l). A good correlation was observed between the method studied and the radiometric method. The method evaluated has suffcient analytical sensitivity to diagnose carnitine deficiencies. The short time period required for sample processing (30 samples in 40min), the simple methodology and apparatus, the ease of personnel training and the low cost of the reagents make this method a good alternative to the classical radiometric method for evaluating serum L-carnitine in clinical laboratories without radioactive installations.


Journal of Automatic Chemistry, Vol. 20, No. 1 (January–February 1998) pp. 23–26

### Automation of a spectrophotometric method for measuring L-carnitine in human blood serum

**Amparo Galan, Anna Padros, Marta Arambarri and Silvia Martin**

*Clinical Biochemistry Service, Hospital Universitario 'Germans Trias i Pujol,' Badalona, Barcelona, Spain*

*A spectrometric method for the determination of L-carnitine has been developed based on the reaction of the 5,5′ dithiobis-(2-nitrobenzoic) acid (DTNB) and adapted to a Technicon RA-2000 automatic analyser (Química Farmacéutica Bayer, S.A.). The detection limit of the method is 13.2 μmol/l, with a measurement interval ranging from 30 to 320 μmol/l. Imprecision and accuracy are good even at levels close to the detection limit (coefficient of variation of 5.4% for within-run imprecision for a concentration of 35 μmol/l). A good correlation was observed between the method studied and the radiometric method. The method evaluated has sufficient analytical sensitivity to diagnose carnitine deficiencies. The short time period required for sample processing (30 samples in 40 min), the simple methodology and apparatus, the ease of personnel training and the low cost of the reagents make this method a good alternative to the classical radiometric method for evaluating serum L-carnitine in clinical laboratories without radioactive installations.*

### Introduction

L-Carnitine (3-hydroxy-4-trimethyl butyric amino acid) is a synthesized quaternary amine from methionine and lysine [1, 2] found in the human liver, brain and kidney [3]. It is an essential cofactor for the transportation of long chain fatty acids through the mitochondrial membrane [4] thereby contributing to the beta-oxidation of the same in the liver, heart and skeletal muscle [5]. It also modulates the intracellular concentration of CoA and acetyl-CoA [6] and eliminates such non-physiologic acyl groups as the benzoic, pivalic and valproic acid [7].

A deficiency of L-carnitine means that the long chain fatty acids will not be oxidized. Clinical manifestation of this dysfunction is presented as myopathy, which may range from simple muscular weakness or slight intolerance to exercise to fatal encephalopathic episodes with hepatic dysfunction or even cardiomyopathy.

Primary L-carnitine deficiencies have been classified into two categories [8, 9]: deficiencies of exclusively muscular localization, and those of systemic involvement. Secondary L-carnitine deficiency, however, is the most common and is associated with pregnancy, malnutrition and cachexia, hepatic cirrhosis, Reye's syndrome, kidney insufficiency with haemodialysis, Fanconi syndrome, prolonged intravenous nutrition, anorexia nervosa, treatments with valproic acid and endocrinologic disorders, etc.

To diagnose these diseases, L-carnitine has to be quantified in peripheral blood and tissues. Several methods have been developed for this: spectrometric [10, 11], radiometric [12, 13], enzymatic [14], fluorometric [15, 16], chromatographic [17] and mass spectrometry [18].

Since the main interest in determining L-carnitine is for the evaluation of deficiencies, the methods used require analytical sensitivity. This condition is difficult to achieve with spectrometric and enzymatic methods, since they often have high coefficients of variation when used at levels close to the detection limit of the method. Nonetheless, these methods are the most appropriate for working in a routine clinical biochemical laboratory, given that they do not require high technology equipment, radioactive installations or highly specialized personnel.

In order to improve analytical sensitivity, precision and accuracy of the spectrometric method for evaluating carnitine, the authors adapted the 5,5′-dithiobis-(2-nitrobenzoic)acid method (DTNB) of Marquis and Fritz [10] to an automatic Technicon RA-2000 (Química Farmacéutica Bayer, S.A.) analyser. The potential of the new method was compared with the classical radiometric method proposed by Cederblad and Lindstedt [13], and modified by Barth *et al.* [19].

### Materials and methods

### *Principle of the method*

The method, which was initially described by Marquis and Fritz [10], is based on the reaction catalysed by the carnitine acetyltransferase (CAT) enzyme (EC 2.3.1.7) acting on L-carnitine:

$$\text{L-carnitine} + \text{acetyl-CoA} \xrightarrow{\text{CAT}} \text{acetyl-carnitine} + \text{coenzyme A}$$

The free coenzyme A combines with the 5,5′-dithiobis-(2-nitrobenzioc) acid forming a fenolate ion spectrometrically measured at 405 nm.

### *Reagents and work solutions*

#### *Reagents*

- 1M Tris HCl, pH 7.8 (Merck cat no. 8382). Stable for at least six months stored between 2 and 8°C.
- 5,5′-Dithiobis-(2-nitrobenzoic) acid (Boehringer Mannheim, cat no. 104477) at a concentration of 12.5 mM in potassium carbonate at 1%, pH 8. Frozen in aliquots; stable for six months stored at −20°C.

0142-0453/98 $12·00 © 1998 Taylor & Francis Ltd

- 15 mM Acetyl-coenzyme A (Boehringer Mannheim cat. no. 101893). Frozen in aliquots; stable for two months at −20°C.
- 50 mM Ethylene diamine tetraacetic acid disodium salt, pH 8 (Titriplex III, Merck cat. no. 8418). Stable for at least six months stored at 4°C.
- 20 U/ml Carnitine acetyltransferase (EC 2.3.1.7) (Boehringer Mannheim cat. no. 103241, specific activity 80 U/mg, 25°C) in phosphate buffer 0.5 M, pH 7.5. Diluted 1:50 in distilled water; stable for six months at 4°C.
- L-Carnitine internal standard: L-carnitine (Boehringer Mannheim cat. no. 836567) of 35, 77, 155, 179, 275, and 310 µmol/L. Frozen in aliquots; stable for at least six months at 20°C.

*Working solutions*

Reagent solution 1: 0.04 mM tris-HCl buffer, 0.05 M 5,5′-dithiobis-(2-nitrobenzoic) acid, 0.06 mM acetyl-coenzme A and 0.5 mM ethylene diamine tetraacetic acid. Prepare immediately before assay.

Reagent solution 2: 0.4 U/mL carnitine acetyltransferase.

*Automated assay*: The working conditions in the RA-2000 analyser were as follows: type of reaction: end-point with sample blank; specimen volume: 30 µl; volume of the first reagent: 350 µL; volume of the second reagent: 50 µL; wavelength: 405 nm; preincubation time: 15 s; Incubation time; 8 min; second reagent addition time: 1 min; work temperature: 37°C.

*Calibration method*: The method was calibrated with an L-carnitine aqueous solution of 155 µmol/l.

*Quality control*: Standards of L-carnitine were processed simultaneously with the problem samples. The assay was considered exact when the values of the standards were within ±10%. Imprecision was controlled with a serum pool.

*Subjects*: To study the reference values of the method, the blood of 100 patients from the Department of Preventive Medicine of the Hospital was used. Blood extraction was carried out after 8 h of fasting. All the subjects in whom the presence of associated diseases, toxic habits or drug ingestion were observed were discarded.

Sixty of these values were used to study the correlation of the method in which serum from 14 pregnant women, 21 patients on haemodialysis, three patients with hepatic cirrhosis, 10 with morbid obesity and 12 undergoing parenteral nutrition were also included.

*Specimens*: The blood samples (5 ml) were extracted by the Vacutainer system in a dry tube without additives. Following retraction of the clot, the samples were centrifuged for 10 min at 3000 rpm. The resulting serum was deproteinized following the procedure described by Rodriguez-Segade *et al.* [20]: approximately 1 ml of serum was kept in a water bath at 100°C for 5 min. After being maintained at −20°C for 40 min the serum was pricked with a needle tip until totally thawed and centrifuged at 15 000 rpm for 15 min. The clear supernatant obtained after centrifugation was the specimen used to determine free carnitine.

*Evaluation of the method*: The analytical interval and the detection limit of the method were evaluated following the directions of the Societé Française de Biologie Clinique [21]. To calculate the analytical interval standard aqueous solutions of L-carnitine were used. The detection limit was established following the processing of 10 specimens of distilled water. With the mean (md) and the standard deviation (SD) the detection limit for an $\alpha$ and $\beta$ risk of 5% was calculated ($Ld = md + K\ SD$).

The recommendations of the European Committee for Clinical Laboratory Standards (ECCLS) were followed for the study of the imprecision and inaccuracy of the method [22].

To determine the analytical recovery, increasing quantities of L-carnitine were added to different aliquots of sera pool. The dilution effect was corrected by adding the same volume of saline solution to aliquot lanes. The reference values of the method were obtained following the recommendations of the Panel of Experts of the IFCC in reference values [23].

*Correlation of the study method with the radiometric method*: L-Carnitine was determined in 120 samples simultaneously by two methods: the study method and a radiometric method. The latter was a modification of the method by Cederblad and Lindstedt [18] proposed by Barth *et al.* [19].

*Statistical methods*: Mean, standard deviation and coefficient of variation for studying the accuracy, imprecision and detection limit. Deming's regression analysis was also used to study the correlation of methods.

To calculate the reference values of the method, the 2.5, 50 and 97.5 percentiles (P2.5, P50, and P97.5) were used.

*Practicability study*: To assess the practicability of the method, the following aspects were taken into account: the installations and apparatus required, training of personnel, time and performance of the assay (preparation of reagents, treatment of the sample, processing of specimen), expiry dates and costs of the reagents. The practicability of the method was compared with that of the radiometric method (taken as the reference).

### Results

*Detection limit and analytical interval.* The detection limit established for the method was 13.2 µmol/l. The analytical interval of the method ranged from 30 to 320 µmol/l. Figure 1 shows the slope of linear regression of the least squares ($y = 0.9x + 6.0; r = 0.98$) established among the theoretical values ($x$) of the six L-carnitine reference standards, processed in triplicate over three consecutive days, and the values found ($y$).

*Imprecision and inaccuracy.* Table 1 demonstrates the within-run and between-run coefficients of variation of the aqueous internal standards of L-carnitine at three concentration levels, as well as those of the serum specimen. The percentages of inaccuracy with respect to the theoretical value (table 2) did not exceed 10%.

*Analytical recovery study.* Analytical recovery of L-carnitine accounted for 89 to 97% (see table 3).

*Reference values.* The reference interval for the whole population studied ranged from 52 to 96.8 µmol/l.

*Table 1. Method imprecision.*

| | *n* | *Within-run CV* (%) | *Between-run CV* (%) |
|---|---|---|---|
| Aqueous L-carnitine solution (35 µmol/l) | 20 | 4.9 | 5.4 |
| Aqueous L-carnitine solution (170 µmol/l) | 20 | 4.6 | 4.9 |
| Aqueous L-carnitine solution (275 µmol/l) | 20 | 1.5 | 4.3 |

*Correlation with the radiometric method.* The slope of the linear regression of least squares between the colorimetric method ($y$) and the radiometric method ($x$) was $y = 1.24x + 8.60$ and linear coefficient of correlation of Pearson was 0.77 (see figure 2). Deming's regression analysis ($b = 1.3528$, $a = 3.969$) shows that the method measures values higher than the radiometric method.

*Practicability of the method.* Table 4 shows the features studied in order to evaluate the practicability of the method in comparison with the classical radiometric method.

### Discussion

The main clinical application of the evaluation of L-carnitine is for evaluating L-carnitine deficiency in human serum or tissues. The enzymatic [14] and spectrometric methods [10] for quantifying this biochemical magnitude generally have low analytical sensitivity for evaluating L-carnitine levels below the reference interval of the method. The coefficients of variation in these ranges are high. The problem is exacerbated with the deproteinization process required for the sample [10, 24], since the treatment with the deproteinization method

*Table 2. Inaccuracy.*

| | | *Within-run* | | *Between-run* | |
|---|---|---|---|---|---|
| *Concentration (theoretical) in µmol/l* | *n* | *Concentration found (µmol/l)* | *Inaccuracy* (%) | *Concentration found (µmol/l)* | *Inaccuracy* (%) |
| 77 | 20 | 72.2 | 6.2 | 74 | 3.9 |
| 155 | 20 | 164.5 | 6.1 | 163 | 5.1 |
| 310 | 20 | 300 | 3.3 | 301 | 2.9 |

*Table 3. Recovery study.*

| *Serum pool (µmol/l)* | *L-carnitine added (µmol/l)* | *Theoretical carnitine concentration (µmol/l)* | *Carnitine concentration found (µmol/l)* | *Recovery* (%) |
|---|---|---|---|---|
| 68.3 | 186 | 254 | 228 | 90 |
| 68.3 | 124 | 192 | 170 | 89 |
| 34.1 | 62 | 96 | 95 | 97 |
| 16.7 | 31 | 47.7 | 46 | 95 |

*Table 4. Practicability of the spectrometric method versus the radiometric method.*

| | *Radiometric method* | *Spectrometric method* |
|---|---|---|
| Type of installation | Radioactive | Conventional |
| Apparatus | ß-Scintillation counter<br>Refridgerated centrifuge | Automatic analyser or spectrometer |
| Personnel training | Requires title of radiosotope manipulator | Relatively simple |
| Response time | 24 h | Approximately 2 h |
| Sample pretreatment | Not required | 50 min |
| Sample processing | Manual: 2 h and posterior radioactivity count | Automatic: 9 min. |
| Reagents | | |
| Reagent preparation | Longer and more difficult than in the radiometry | Relatively simple |
| Expiry date of reagents | Long | Six months |
| Cost of reagents | | 2.3-fold lower than radiometric method |

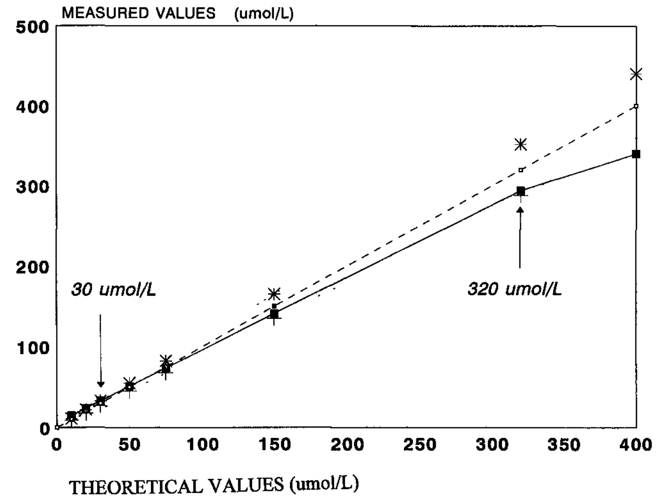

*Figure 1. Analytical interval: reference line* ( —■— ) y = bx (b = 1) *and 2 standard deviations of reference line* y = x (+), (✱). *The points from 30 to 320* μmol/l *on the experimental line* (y = 0.9 x + 6) (-■-) *cross either directly or at least in the range of their 2 standard deviations the reference line.*

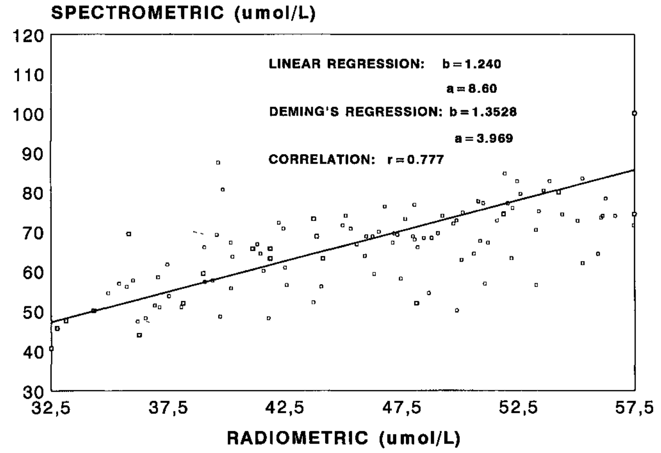

*Figure 2. Correlation between the radiometric and the colorimetric method.*

often leads to an even greater decrease in carnitine concentration.

With the proposed method the analytical sensitivity at low levels markedly improved, the between-run coefficient of variation for a concentration of 35 μmol/l was 5.4%. This concentration is lower than the low interval of the method reference value (52 μmol/l) and is close to the 13.2 μmol/l of the detection limit. The improvement achieved is due to the automatization process of the spectrometric method of the 5,5′-dithiobis-(2-nitrobenzoic) acid, and also to the use of thermic deproteinization described by Rodriguez-Segade *et al.* [20] which, among other advantages, avoids any dilution of the sample. On adapting the technique by Marquis and Fritz [10], an automatized method is able to markedly save the volume of the deproteinized sample. Thirty microlitres were used versus the 1 ml required by the original technique.

From a practical point of view, the adapted method has advantages over the radiometric method. The results were obtained quickly, with 30 deproteinized samples being processed in 40 min. The cost of the reagents is 2.3-fold less than that of the radiometric method. Moreover, training of personnel for performing this technique is relatively simple and the apparatus required are generally found in a conventional clinical laboratory. The radiometric methods used as reference methods (because of their greater analytical sensitivity) present the inconveniences of using radioisotopes requiring radioactive installations which not all clinical laboratories possess. Thus, the evaluation presented demonstrates excellent applicability, particularly for laboratories without radioactive installations.

The method developed and presented in this study has sufficient analytical sensitivity to evaluate defiencies in serum carnitine. This method may substitue the classical radiometric method and should be considered in clinical laboratories without radioactive installations.

### References

1. Tanphaichitr, V. and Broquist, H. P., 1973, *Journal of Biological Chemistry*, **248**, 2176.
2. Wolf, G. and Berger, C. R. A., 1961, *Arch. Biochem. Biophys.*, **92**, 360
3. Rebouche C. J. and Engel, A. G., 1980, *Biochimica Biophysica Acta*, **630**, 22.
4. Stumpf, D. A., Parker, W. D. and Angelini, C., 1985, *Neurology*, **35**, 1041.
5. Pande, S. V. and Parvin, R., 1980, *Journal of Biological Chemistry*, **255**, 2994.
6. Chalmers, R. A., Roe, C. R., Stacey, T. E. and Hoppel, C. L., 1984, *Pediatric Research*, **18**, 1325.
7. Millintong, D. S., Bohan, T. P., Roe, C. R., Yergey, A. L. and Liberato, D. J., 1985, *Clinica Chimica Acta*, **145**, 69.
8. Dipalma, J.,1988, *American Family Physician*, **38**, 243.
9. Siliprandi, N., Sartorelli, L., Ciman, M. and Di Lisa, F., 1989, *Clinica Chimica Acta*, **183**, 3.
10. Marquis, N. R. and Fritz, L. B., 1964, *Journal of Lipid Research*, **5**, 184.
11. Seccombe, D., Dodek, P., Frohlich, J., Hahn, P., Skala, J. and Campbell, D., 1976, *Clinical Chemistry*, **22**, 209.
12. Barns, R., Bowling, F., Brown, G., Clague, A. and Thompson, A., 1991, *Clinica Chimica Acta*, **197**, 27.
13. Cederblad, G. and Lindstedt, S., 1972, *Clinica Chimica Acta*, **37**, 235.
14. Wieland, O. H., 1985, In *Methods of Enzymatic Analyses*, Ed. Bergmeyer, H. U. (Weinheim: VCH Verlagsgesellschaft).
15. Matsumoto, K., Yamada, Y., Takahashi, M., Todoroki, T., Mizoguchi, K., Misaki, H. and Yuki, H., 1990, *Clinical Chemistry*, **36**, 2072.
16. Maehara, M., Kinoshita, S. and Watanabe, K. , 1988, *Clinica Chimica Acta*, **171**, 311.
17. Weading, G. P., Mills, G. A. and Walker, V., 1988, *Annals of Clinical Biochemistry*, **25**, 233s.
18. Kodo, N., Millington, D., Norwood, D. and Roe, C. 1989, *Clinica Chimica Acta*, **186**, 383.
19. Barth, P. G., Scholte, H. R., Berden, J. A. *et al.*, 1983, *Journal of the Neurological Sciences*, 327.
20. Rodriguez-Segade, S., Alonso de la Peña, C., Paz, J. M. and Del Río, R., 1985, *Clinical Chemistry*, **31**, 754.
21. Societé Française de Biologie Clinique, 1986, *Ann. Biol. Clin.*, **44**, 686.
22. European Commitee for Clinical Laboratory Standards, 1986, *Guidelines for the Evaluation of Analyzers in Clinical Chemistry*, ECCLS Document.
23. International Federation of Clinical Chemistry, 1987, *Clinica Chimica Acta*, **170**, s13.
24. Fishlock, R. C., Bieber, L. L. and Snoswell, A. M., 1984, *Clinical Chemistry*, **30**, 316.

